# The modified suture-bridge technique for treating avulsion fracture of minors tibial eminence of anterior cruciate ligament: a retrospective study

**DOI:** 10.1186/s13018-024-04914-6

**Published:** 2024-07-18

**Authors:** Yimin Du, Zhaojun Wang, Shaojun Wu, Peng Zhou, Zheng Li, Jinghong Yang, Jun Zhong, Zhong Li, Juncai Liu

**Affiliations:** 1 Department of Orthopaedics, Stem Cell Immunity and Regeneration Key Laboratory of Luzhou, The Affiliated Hospital of Southwest Medical University,, Sichuan Provincial Laboratory of Orthopaedic Engineering, Luzhou, 646000 Sichuan People’s Republic of China; 2 Dazhou Dachuan District People’s Hospital (Dazhou Third People’s Hospital), Dazhou, 635700 Sichuan People’s Republic of China; 3 Department of Trauma and Reconstructive Surgery, RWTH Aachen University Hospital, 52074 Aachen, Germany

**Keywords:** ACL tibial avulsion (eminence) fracture, Suture bridge, Adolescents, Arthroscopy, Clinical effectiveness

## Abstract

**Purpose:**

This study aimed to evaluate the clinical and radiological outcomes of modified suture-bridge technique fixation for anterior cruciate ligament (ACL) tibial avulsion fracture.

**Method:**

Minors who underwent arthroscopic reduction and modified suture bridge fixation of ACL tibial avulsion fracture between January 2018 and January 2022 were retrospectively analyzed. Postoperative MRI and X-ray examinations were performed to evaluate the presence of epiphyseal plate injury and fracture healing. Moreover, KT-1000 side-to-side difference, Lachman test, range of motion (ROM), the subjective Knee score of the International Knee Documentation Committee (IKDC), Lysholm Knee score, and Tegner activity grade score were evaluated preoperatively and at the minimum 1-year follow-up visit.

**Results:**

A total of 16 participants met the inclusion criteria. They had a mean age of 12.6 years (range, 9–16 years); mean time to surgery, 6.9 days (range, 2–13 days) and had a minimum of 12 months clinical follow-up (mean, 25.4 months; range, 12–36 months) after surgery. Postoperative radiographs and MRI showed no injury to the epiphyseal plate, optimal reduction immediately after the operation, and bone union within three months in all patients. All of the following showed significant improvements (pre- vs. postoperatively): mean KT-1000 side-to-side difference (8.6 vs. 1.5; *p* < 0.05), Lachman tests (2 grade 9 and 3 grade 7 vs. 0 grade 12 and 1 grade 4; *p* < 0.05), IKDC subjective score (48.3 vs. 95.0; *p* < 0.05), mean Lysholm score (53.9 vs. 92.2; *p* < 0.05), mean Tegner activity score (3.2 vs. 8.3; *p* < 0.05) and mean ROM (42.9°vs 133.1°; *p* < 0.05).

**Conclusion:**

Arthroscopic reduction and modified suture bridge fixation for ACL tibial avulsion fracture is a dependable and recommended treatment that can effectively restore the stability and function of the knee and is worthy of clinical promotion.

## Background

Fractures of the tibia eminence, first described by Poncet in 1875 [1], are serious injuries involving bony avulsion of the anterior cruciate ligament (ACL) from the intercondylar eminence [2, 3] and are a significant intra-articular injury in the pediatric knee [4, 5]. However, at times, they can also occur in adults, whose primary cause is high-energy trauma such as falls, skiing, and motor vehicle accidents [6, 7], and the prevalence in young adults has been increasing [8].

Treatment techniques include surgical open reduction with internal fixation (ORIF) and arthroscopic approaches with internal fixation (ARIF) [9]. The arthroscopic reduction and internal fixation techniques can use a variety of fixation methods, such as metal screws [10], sutures [11–13], and suture anchors [14]. Of these methods, most biomechanical studies of porcine or human bone show that suture fixation is superior to screw fixation [15]. Although there are various biochemical comparative studies of arthroscopic tibial eminence fracture fixation methods, the best choice of technique remains controversial [16–19]. Although most adult procedures equally apply to children, epiphyseal integrity is critical. To minimize the risk of growth retardation and femoral and tibial deformities, surgeons should minimize the risk to the epiphyseal plate during transposal ACL reconstruction [19]. The effect of drilled screw fixation on immature tibial epiphyses may lead to growth disturbances, as has been demonstrated in several clinical studies and animal experiments [20]. In recent years, due to the rotator cuff suture bridge technique, some researchers have developed a new treatment method: the arthroscopic suture bridge technique. The suture bridge technique has a higher failure load compared to the traditional screw and suture fixation technique [18, 21]. It does not pass through the tibial metaphysis, all of which are good surgical indications for pediatric tibial intercondylar ridge fractures. However, in the current application of the suture bridge technique, there are problems such as poor firmness due to fixation of external rows of anchors in the intra-articular cancellous bone, loss of suture tension due to manual suturing, and increased operative complexity and cost due to excessive use of intra-articular anchors. Therefore, improving the existing suture bridge technique has become an urgent problem.

This study aimed to evaluate the clinical outcome of the modified arthroscopic suture bridge technique for tibial eminence fracture in children and adolescents and assess knee stability and function. We hypothesized that the arthroscopic suture bridge technique for tibial eminence fracture in children and adolescents could restore keen stability and function.

## Methods

### Patients

This study was approved by the Ethics Committee of the Affiliated Hospital of Southwest University. A retrospective chart review of children and adolescent tibial intercondylar eminence fracture patients treated at our institution between January 2018 and January 2022 was conducted. The inclusion criteria were displaced tibial eminence fractures (Meyer-McKeever type II, III, and IV) in underage patients whose epiphyseal line is not closed. All tibial eminence fractures are classified at least as grade II on Meyers-Meever grading. Imaging, including X-ray, CT, and MRI, was performed before surgery and arthroscopic confirmation of undamaged ACL parenchyma. Surgery was performed within three weeks at least after acute avulsion fracture, and patients were followed up at a minimum of 12 months.

Patients who met any of the following criteria were excluded: (a) concomitant multiple ligament ruptures such as posterior cruciate ligaments or collateral ligaments; (b) tibial plateau fracture; (c) keen joint hypermobility (Beighton score exceeds (5); d) less than 12 months follow-up; and patients who do not want to co-operate.

### Surgical technique

**Fig. 1:**
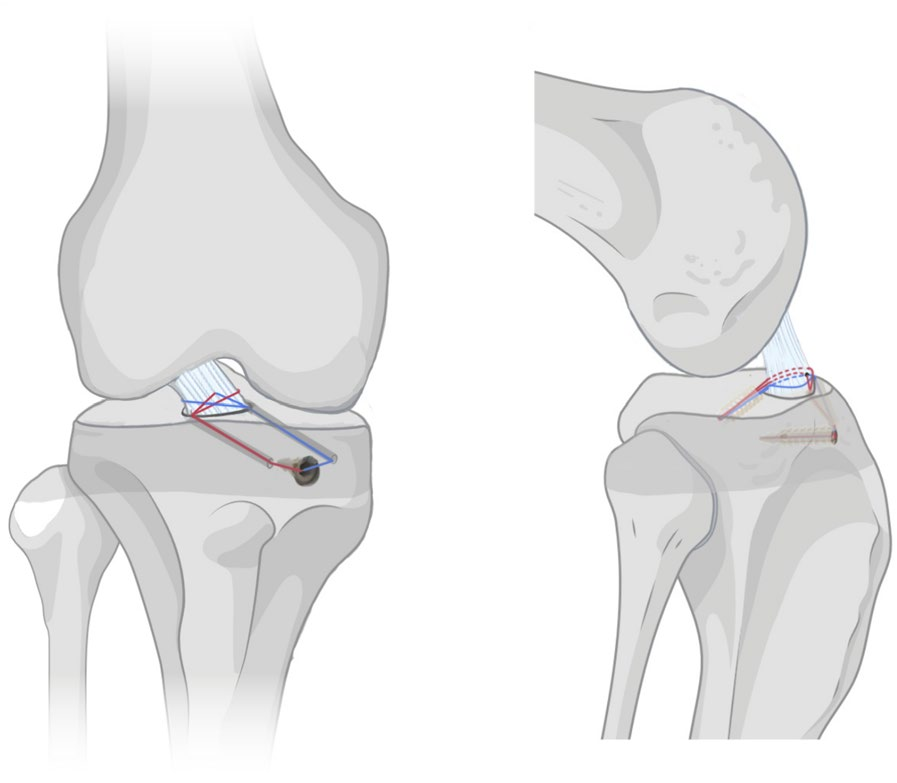
Schema showing the procedure for the modified suture-bridge technique

The same orthopedic surgeon operated on all surgical procedures within three weeks after the injury. Under Combined intravenous anesthesia, patients are positioned supine on the operating table. A repeat anterior drawer test proved positive. An airbag tourniquet was applied to the proximal end of the affected limb with a pressure setting of limb occlusion pressure (LPO) + 50 mmHg. The sterile drapes were spread after conventional povidone-iodine disinfection in the operation area. Using standard anterolateral and anteromedial approaches to expose the knee joint, standard knee arthroscopy was performed to examine the joint thoroughly, and concomitant meniscus lesions were treated (Fig. 1).

**Fig. 2:**
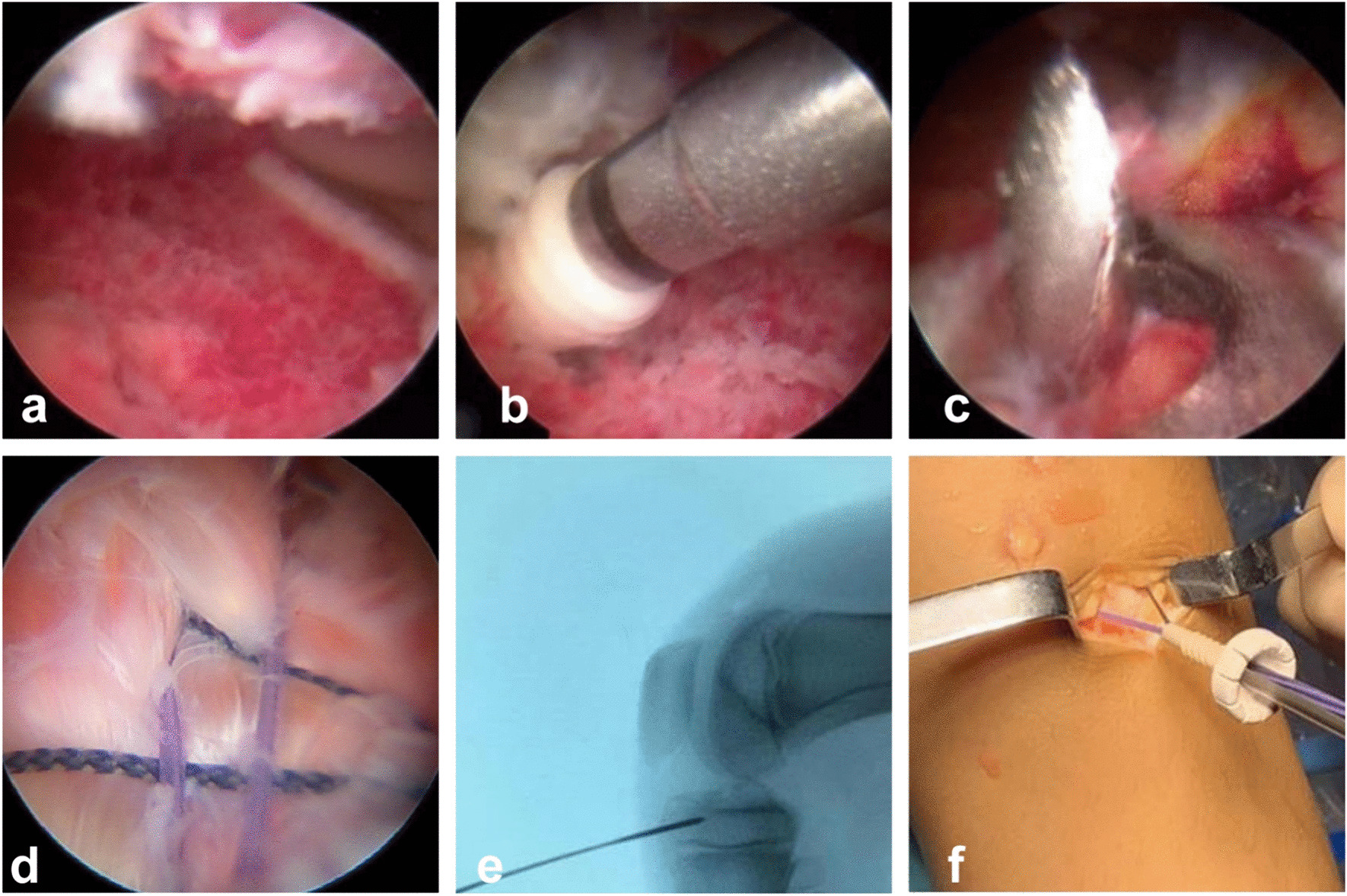
Female patient, nine years old, ACL tibial stop avulsion fracture of the left knee. **a** Arthroscopic demonstration of the broken end of the avulsion. **b** One 2-Orthocord suture anchor was screwed into the posterior, posterolateral portion of the metaphyseal bed. **c** Kirschner pins are drilled medially and laterally in the anterior portion of the metaphyseal bed to create a tunnel of the bone. **d** The sutures were crossed to form a suture bridge to secure the avulsed bone block. **e** Tunnel positioning above the metaphysis under C-arm fluoroscopy. **f** Sutures are secured by external rows of anchor at the outer portion of the tunnel

Place a motorized shaver to clean up the blood clot at the fracture end to enhance visualization, and use a probe to loosen the soft tissues at the embedded fracture end until the fracture end is exposed. Under intraoperative C-arm fluoroscopic, with a syringe needle locating the metaphyseal plane on the patient's body surface, two bone tunnels were made in the tibia by drilling holes above the metaphyseal plane, using an ACL locator and two 2.0 mm Gram pins, of which the outer openings on the medial side of the tibial tuberosity and above the metaphyseal plate at a distance of about 1 cm from each other, as the inner openings of the two tunnels were located at the anteromedial and anterolateral sides of the fracture block, respectively. A reamer was used to create a pilot hole slightly outside the center of the posterior border of the bone bed, and a 4.5-mm internal row anchor (4.5 mm HEALIX ADVANCE BR anchors, Johnson & Johnson) with a 2Orthcord suture was placed obliquely downward at approximately 45° in a zig-zag pattern to the two inner tunnel ports. The reamer drills a pilot hole in the medial tibial tuberosity in an isosceles triangle with the outer portions of the two tunnels. One end of each suture was passed through the parenchyma of the ACL, and the other two were wrapped around the base of the ACL and crossed anteriorly in a double "8" pattern. The fracture block was arthroscopically repositioned with the sutures, the four strands of suture that were externally drawn out of the joint were tightened, and a 4.5-mm external row anchor (4.5 mm HEALIX ADVANCE BR anchors, Johnson & Johnson) was squeezed into place to complete the fixation. The surgical procedure is shown in Fig. 2.

### Postoperative rehabilitation

Routine postoperative antibiotics were administered for 24 h to prevent infection, and cold compress, detumescence, and analgesia were applied after surgery. After the surgery, each patient was immobilized with a full-extension adjustable knee brace. Quadriceps strengthening, isometric exercises, ankle pump training, and patellar movement exercises were encouraged, along with straight-leg raises after awakening from anesthesia. Light passive knee flexion on postoperative day 1; non-weight bearing activities on day 2 with the protection of a brace. After two weeks, the patient could gradually start weight-bearing and strengthen the functional exercise. At four weeks, the knee could be flexed up to 90° and fully weight-bearing. Most importantly, no strenuous exercise for three months. At three months, with the follow-up X-rays indicating fracture healing, patients performed low-intensity, non-confrontational exercises while removing the brace. At six months, all patients could start oppositional exercise. The functional exercise was guided by regular follow-up at months 1, 3, 6, and 12 postoperatively.

### Clinical outcome assessment

All the subjects underwent clinical and radiographic assessments during the follow-up. Postoperatively, at three months, 6 months, nine months, one year, and then annually, radiographs and magnetic resonance imaging were obtained. At preoperative and the last follow-up, all the patients were evaluated using anterior tibial translation (ATT) side-to-side difference, and the Lachma range of motion was assessed actively and passively with a goniometer. Knee function was evaluated by the Lysholm and International Knee Documentation Committee (IKDC) scores, and the Tegner scale rated the activity level before the injury and at follow-up. Patients were also evaluated according to the IKDC knee examination form, and their knees were graded as normal (grade A), nearly normal (grade B), abnormal (grade C), or severely abnormal (grade D).

### Imaging evaluation

The long leg alignment views were crucial and significantly required to determine the presence of angular deformity or growth arrest and the condition of bone tunnels and internal fixations. In addition, an MRI examination was needed to show the tension and continuity of the graft.

### Statistical analysis

All statistical analyses were performed with IBM SPSS Statistics software (version 17.0; IBM, Armonk, NY). A paired t-test or rank test by the assumption of normality and chi-squareness was conducted to test for all continuous variables (e.g., Keen range of motion, Tegner score difference, Lysholm score, and IKDC score). Categorical variables (Lachman's scale) were tested using the chi-square test or Fisher's exact test. Test level *α* = 0.05.

## Results

A total of 16 participants met the inclusion criteria. They had a mean age of 12.6 years (range, 9–16 years) and a mean time to surgery of 6.9 days (range, 2–13 days). The cause of injury was related to sports in 10 patients, a simple fall in 2, and a car accident in 4. The demographic and injury data of the patient population are analytically presented in Table 1.

**Table 1:** Demographic and injury data of patient population

Patient no	Age(yr)	Sex	Cause of injury	Type of Fracture	Concomitant injuries	Time to surgery (d)
1	12	M	Traffic accidents	IV	LM	3
2	13	M	Sport activities	III	–	9
3	14	F	Sport activities	III	LM	4
4	11	M	Fall	III	–	6
5	12	M	Sport activities	II	–	4
6	14	M	Sport activities	III	–	5
7	10	F	Traffic accidents	III	–	11
8	15	M	Sport activities	II	LM	9
9	16	M	Sport activities	III	–	7
10	11	F	Fall	III	–	3
11	13	M	Traffic accidents	III	LM	11
12	12	F	Sport activities	II	–	13
13	14	F	Sport activities	II	–	8
14	14	M	Traffic accidents	III	LM	2
15	9	F	Sport activities	III	–	5
16	12	M	Sport activities	II	–	11

*F* female, *LM* lateral meniscus tear, *M* male

The mean time of surgery was 82.9 min (range 65–100 min). All incisions healed to stage I, and no postoperative complications such as infection, vascular injury, or nerve injury were observed.

**Fig. 3:**
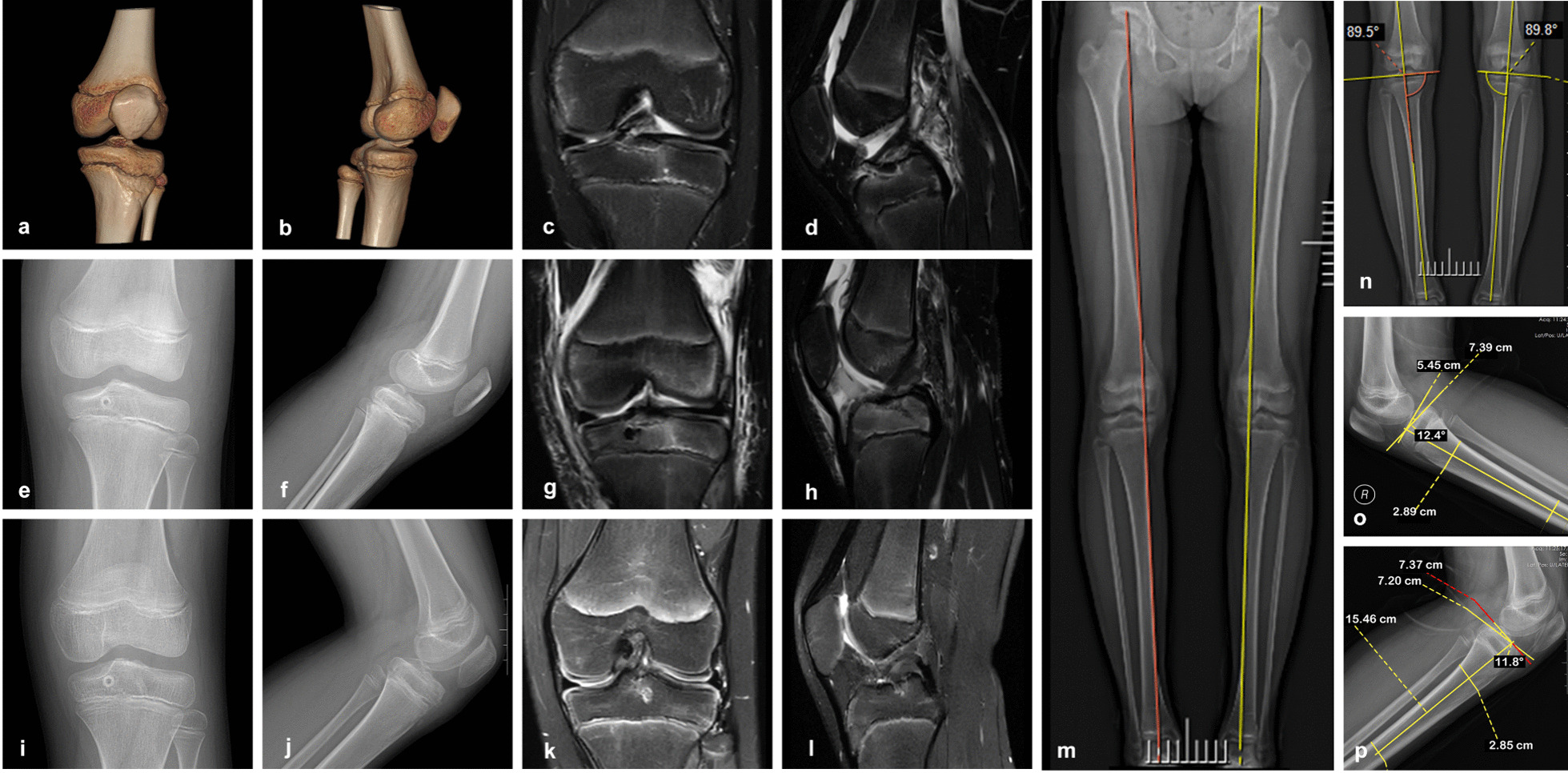
Female patient, nine years old, ACL tibial eminence avulsion fracture of the left knee. **a**–**d** Preoperative and images showing the type III tibial intercondylar eminence avulsion fractures. **a**, **b** Preoperative MRI. **c**, **d** Preoperative CT + 3D reconstruction. **e**–**l** Postoperative and follow-up images showed a good reduction of displaced tibial eminence fracture fragments and the restoration of the displaced fracture fragments. **e**, **f** X-ray on the third postoperative day. **g**, **h** MRI on the third postoperative day **i**, **j** X-ray on the first year postoperative. **k**, **l** MRI on the first year postoperative. **m**–**p** Postoperative and last follow-up X-ray images showed no difference in the length of the lower limbs and no angular deformities. **m** Long leg alignment on the third postoperative year showing the length of the lower limbs. **n** Long leg alignment on the third postoperative year showing the medial proximal tibial angle (MPTA). **o**, **p** Postoperative x-ray showing posterior tibial slope (PTS)

All the patients were followed for a mean of 18.7 months (range, 12–36 months). A satisfactory fracture reduction was confirmed through the last time follow-up X-ray image and MRI. Fractures reached bony healing at three months postoperatively, with good ACL tension and alignment (Fig. 3).

### Side-to-side difference

At the final follow-up, a side-to-side difference of anterior tibial translation was significantly decreased from 8.60 mm (range 5.5–11.6) preoperatively to 1.5 mm (range 0.3–2.7) in the last follow-up (*p* < 0.05).

### Lachman tests

The Lachman test identified 9 patients in grade 2 and 7 patients in grade 3 preoperatively, and postoperatively, there were 12 in grade 0 and 4 in grade 1 (*p* < 0.05).

### IKDC evaluation

The mean IKDA scores were significantly improved from 48.3 (range 38.9 to 57.7) at the time of surgery to 95.0 (range 91.4–98.6) at the final follow-up (*p* < 0.05).

### Lysholm knee scores

The Lysholm knee scoring system was used to analyze subjective symptoms. The mean preoperative Lysholm score was 53.9 (range 47.4–60.4); the mean postoperative Lysholm score was 92.2 (range 85.7–98.7). Lysholm scores significantly differed between preoperative evaluation and final follow-up (*p* < 0.05).

### Tegner activity level

The mean preoperative Tegner scores were 3.2 (range 1.6–4.8), respectively. The mean postoperative Tegner score was 8.3 (range 6.9–9.7). Improvement from preoperative to postoperative values was statistically significant (*p* < 0.05) (Table 2).

**Table 2:** Functional evaluation and stability testing

	Preoperative	Postoperative	
Side-to-side difference (mm)	8.6 ± 3.0	1.5 ± 1.2	*t* = 8.6433 *p* = 0.000
Lachman test			
0	0	12	*Z* = 5.047 *p* = 0.000
I	0	4	
II	9	0	
III	7	0	
IKDC Evaluation	48.3 ± 9.4	95.0 ± 3.6	*t* = 18.480 *p* = 0.000
Lysholm Knee Scores	53.9 ± 6.5	92.2 ± 6.5	*t* = 16.624 *p* = 0.000
Tegner Activity Level	3.2 ± 1.6	8.3 ± 1.4	*t* = 9.419 *p* = 0.000
ROM(°)	42.9 ± 21.3	133.1 ± 2.4	*t* = 16.837 *p* = 0.000
MPTA(°)		89.15 ± 0.6	
PTS (°)		10.65 ± 1.61	

### Range of motion

The mean knee joint ROM showed significant improvement, increasing from 42.9° (range 21.6°–64.2°) pre-surgery to 133.1° (range 130.7°–135.5°) at the last follow-up postoperatively (*p* < 0.05).

## Discussion

This retrospective study analyzed the clinical outcomes of arthroscopic reduction and trans-epiphyseal tunnel suture-bridge fixation in the treatment of avulsion fractures of the tibial attachment of ACL in children and adolescents. The follow-up period ranged from 12 to 36 months. The results showed that the fixation method was secure and dependable, and the clinical outcomes were satisfactory.

Due to the immaturity of the tibial epiphysis, children are more likely to experience ACL avulsion fractures when subjected to external forces, as compared to adults [4]. Such avulsion fractures disrupt anterior stability while potentially affecting the intercondylar ridge and other parts of the tibial plateau. Keiser et al. [20] found that excessive flexion and rotation were the most common injury mechanisms of ACL avulsion fractures, with over 50% of the included 16 patients being skeletally immature.

Adeinto et al. [7] reported a retrospective study of 83 patients demonstrated that falls, traffic accidents, and sports activities were prevalent causes of such injuries. The findings are consistent with the type of damage discussed in our research.

According to the revised Meyers-Mckeever [15, 23] classification by Zaricznyj [24, 25], tibial avulsion fractures are categorized into four types. The management of type I fractures normally involves conservative treatment [12]. For type II fractures, both surgical and non-surgical methods are widely used, and the optimal treatment remains a subject of debate [22]. Most type III/IV fractures are not reducible due to soft tissue entrapment, and these situations drive surgical treatments, including ARIF and ORIF [9]. ARIF has the advantages of minimal invasion, less post-operative pain, early rehabilitation, and lower risk of knee fibrosis and infection compared to ORIF [26–29]. Additionally, Feucht et al. [30] observed the incidence and injury characteristics of meniscus injuries in children and adolescents undergoing treatment for ACL tibial avulsion fractures. The study revealed that approximately 40% of patients experienced meniscus injuries, among which the most commonly affected site was the posterior horn of the lateral meniscus. In our study, 5 out of the total 16 patients (31%) experienced the lateral meniscus injury. This finding is consistent with Feucht's previous reports. ARIF is superior to ORIF in the treatment of these intra-knee-associated injuries (meniscal or osteochondral lesions), and it is the preferred option to control and drive the reduction in the fragment [6]. Based on the above advantages, ARIF has gradually become the most common technique for treating ACL avulsion fractures over the past few decades.

The objective of surgical treatment is to allow early mobilization of the knee joint, minimize the risk of fibrosis, and accelerate functional recovery through anatomical reduction of the fracture fragment and rigid internal fixation. Due to its significant clinical efficacy in the arthroscopic treatment of rotator cuff tears and fractures of greater tuberosity of the humerus, the suture bridge technique has been gradually introduced to the fixation of ACL avulsion fractures in recent years and has achieved good clinical results [31–34]. In traditional suture bridge fixation, one or two suture anchors are placed behind the fracture bed. After the fracture is reduced, the sutures are threaded through or around the ACL on the surface of the fragments and finally fixed to the external anchors placed on the anteromedial and anterolateral sides of the fracture bed [35, 36]. Previous biomechanical trials have conducted comparisons among three different fixation methods, including cannulated screw fixation, suture fixation, and suture anchor fixation: Sawyer et al. [21] demonstrated that the utilization of the suture bridge fixation technique with suture anchors for treating ACL avulsion fractures resulted in a significantly higher ultimate failure load compared to conventional cannulated screw and suture fixation. Li et al. [18] studies have demonstrated that suture bridge fixation resulted in less displacement of the fracture than conventional suture fixation under cyclic load tests. In clinical practice, compared with traditional screw fixation, suture bridge technology has the following advantages. Firstly, the technique is not constrained by the size of the fracture fragments and is particularly suitable for stabilizing small or comminuted fracture fragments. Secondly, it prevents re-fracture of the fracture fragments caused by excessive screw compression or the impact of the protruding nut on the intercondylar fossa due to insufficient compression. Thirdly, it eliminates the need for a subsequent surgical procedure, resulting in reduced patient costs and minimizing the trauma associated with additional operations. Last but not least, it reduced the occurrence of epiphyseal injuries in adolescents. Compared to the figure-of-8 suture fixation, the suture-bridge fixation represents a planar fixation method. By employing the bridge fixation, the fracture fragment is uniformly compressed flatly, ensuring close contact and pressurization between the fragment and bone bed. This technique mimics tension band fixation and enhances both contact area and pressure while also simplifying the procedure by eliminating the need for knotting. The suture fixation involving two tunnels anterior to the bone fragment may result in an uneven distribution of force on the fragment. In clinical practice, it has been observed that the bone fragment tends to become posteriorly warped and displaced, potentially impacting fracture healing and leading to residual ACL laxity after operation.

However, the traditional suture-bridge fixation exhibits certain limitations, including inconsistent biomechanical fixation and limited surgical space within the joint cavity of children and adolescents, posing challenges in anchor placement and high costs. The trans-epiphyseal tunnel suture bridge fixation used in this study is one of the suture bridge fixation methods. It has the advantages of suture bridge fixation, in which the sutures originating from an internal suture anchor positioned at the posterior edge of the fracture bed are guided out through a tunnel and secured with an external anchor located on the medial side of the tibial tubercle. Compared with the traditional suture bridge fixation, this new technique has the following advantages. Firstly, The external anchors fixed in the medial tibial tubercle cortical bone provide stronger holding power compared to cancellous bone in the joints, thereby theoretically achieving higher biomechanical strength of fixation. Additionally, this technique also eliminates the tension loss caused by traditional manual suture knots. Secondly, The two internal tunnel openings located at the anterior edge of the bone bed effectively replaced the external anchors utilized in traditional suture bridge techniques, which not only saved the cost of fixed materials but also addressed the challenges associated with anchor placement and suture tightening within the limited operating space of children and adolescent joint cavities. In terms of postoperative anterior stability, the KT-1000 difference was measured at 1.5 ± 1.2 mm, with only one patient showing a difference greater than 3 mm; the Lachman test yielded weakly positive results in only 2 cases. As shown in Table 2, the post-operative IKDC, Lysholm, and Tegner scores reached 95.0 ± 3.6, 92.2 ± 6.5, and 8.3 ± 1.4 (*p* < 0.05), demonstrating statistically significant differences between the groups. Additionally, the joint range of motion was restored.

The following points should be paid attention to during the operation: 1. Because ACL avulsion fracture is often accompanied by soft tissue injuries such as meniscus, the joint cavity should be fully explored by arthroscopy, and the related combined injuries should be treated in time; 2. Before performing fracture reduction, it is essential to thoroughly clean the blood clots between the fractured ends and release any incarcerated soft tissue. This will facilitate precise reduction during arthroscopy; 3. The tunnels should be positioned superior to the epiphyseal plate to prevent iatrogenic injury to the epiphysis. The location of the epiphyseal plate on the body surface can be determined using a syringe needle with guidance from the C-arm during surgery. The regular follow-up radiography was concurrently conducted to assess the presence of any deformities in the epiphyseal plate. The PTS measurement was (10.65 ± 1.61)°, while the MPTA measurement was (89.15 ± 0.6)°. No evident shortening or angulation deformity was observed.

This study also has the following limitations. Firstly, it is a retrospective analysis that lacks a control group and has a small sample size. Secondly, the postoperative KT-1000 and Lachman tests were performed without anesthesia, potentially impacting the accuracy of the results. Lastly, further investigation is needed to determine whether suture crossing the ACL affects its blood supply.

## Conclusion

Treatment of tibial ACL avulsion fractures by arthroscopic suture bridge technique is a successful technique to restore tibial avulsion injuries of the ACL with well-documented radiographic healing, good clinical outcomes, and low complication rates.

## Abbreviations

ACL: Anterior cruciate ligament
ACLR: Arthroscopic anterior cruciate ligament reconstruction
ROM: Range of motion
VAS: Visual analog scale
IKDC: International Knee Documentation Committee
MRI: Magnetic resonance imaging
